# Studying protein interactions in human synapses using array tomography

**DOI:** 10.1002/alz.094928

**Published:** 2025-01-09

**Authors:** Kristjan Holt, Lewis W. Taylor, Robert I. McGeachan, Elizabeth Simzer, Martí Colom‐Cadena, Érika Sánchez‐Aced, Declan King, Caitlin Davies, Mary E. Hamby, Mathew H. Horrocks, Alberto Lleo, Claire S. Durrant, Tara L. Spires‐Jones

**Affiliations:** ^1^ Centre for Discovery Brain Sciences at the University of Edinburgh, Edinburgh, Scotland United Kingdom; ^2^ UK Dementia Research Institute at the University of Edinburgh, Edinburgh, Scotland United Kingdom; ^3^ Memory Unit, Department of Neurology, Institut de Recerca Sant Pau ‐ Hospital de Sant Pau, Universitat Autònoma de Barcelona, Barcelona, Spain. Centro de Investigación Biomédica en Red en Enfermedades Neurodegenerativas (CIBERNED), Madrid, Spain, Barcelona, Barcelona Spain; ^4^ Cognition Therapeutics, Inc, Pittsburgh, PA USA; ^5^ Institute for Regeneration and Repair (IRR) at The University of Edinburgh, Edinburgh, Scotland United Kingdom; ^6^ School of Chemistry, The University of Edinburgh, Edinburgh United Kingdom

## Abstract

**Background:**

Synapse loss represents the closest correlate of cognitive decline in Alzheimer’s Disease (AD). Standard microscopy, due to increased diffraction of light with tissue depth, imposes a limit on axial resolution extending to ∼ 700nm. *Array tomography (AT)*, developed by Micheva & Smith (2007), extends this axial limit via physical sectioning of resin‐embedded tissue into ribbons of 70nm contiguous sections that are serially imaged and reconstructed into 3D volumes; thus, allowing for nanometric synaptic puncta to be resolved at the mesoscale. Our group has, for the past decade, adapted AT to investigate synapse loss in human post‐mortem brain tissue from people who died with Alzheimer’s disease; other neurodegenerative diseases, and control subjects. We have published recent developments to AT that further improve X‐Y resolution, permitting in‐depth investigation of synapse‐protein interactions. Highlighted here are Förster Resonance Energy Transfer (FRET), Reconstruction Microscopy (dsSTORM) and DNA‐PAINT to assess interactions between Aβ, tau and protein binding partners, at the synapse*. This work was funded by Cognition Therapeutics, Medical Research Council (MRC), UK Dementia Research Institute (UKDRI) and Race Against Dementia*.

**Method:**

Fixation, embedding, sectioning and immunostaining are detailed in work published previously by Kay *et al.* (2013). Advances to AT presented here include FRET, dsSTORM, DNA‐PAINT, and investigation of synaptic composition in organotypic human brain slices from surgical resections challenged with disease‐relevant proteins. Further, we report advances in our image analysis pipeline with scripts available to use on https://github.com/Spires‐Jones‐Lab.

**Result:**

FRET‐AT has been used to profile over 1 million individual synaptic puncta in the temporal cortex and generates sufficient signal to indicate molecular interaction between Aβ and putative binding partners including TMEM97 in control (n = 9) and Alzheimer’s disease (n = 11) human brain tissue. FRET‐AT also suggests interaction between p‐tau Ser356 and synaptic terminals (n = 5/group). This, coupled with evidence of synaptic tau from dsSTORM/DNA‐PAINT, provides indirect evidence of disease progression via tau‐synapse interaction.

**Conclusion:**

AT can be combined with other methodologies, allowing for in‐depth investigation of synapse‐protein interactions at a scale larger than afforded by electron microscopy (EM). This provides important insight into neurodegenerative disease and presents targets for potential therapeutics disrupting protein‐protein interactions.

